# Liposome Formulation for Tumor-Targeted Drug Delivery Using Radiation Therapy

**DOI:** 10.3390/ijms231911662

**Published:** 2022-10-02

**Authors:** Amanda J. Stolarz, Bijay P. Chhetri, Michael J. Borrelli, Samir V. Jenkins, Azemat Jamshidi-Parsian, Joshua H. Phillips, Daniel Fologea, Jay Gandy, Robert J. Griffin

**Affiliations:** 1 Department of Pharmaceutical Sciences, University of Arkansas for Medical Sciences, Little Rock, AR 72205, USA; 2 Rejuvenics Technologies, LLC, Perryville, AR 72126, USA; 3 Department of Radiology, University of Arkansas for Medical Sciences, Little Rock, AR 72205, USA; 4 Department of Radiation Oncology, University of Arkansas for Medical Sciences, Little Rock, AR 72205, USA; 5 Department of Physics and Biomolecular Sciences Graduate Program, Boise State University, Boise, ID 83725, USA; 6 Department of Pharmacology & Toxicology, University of Arkansas for Medical Sciences, Little Rock, AR 72205, USA

**Keywords:** liposome, tumor-targeted, radiation, tumor, drug delivery, chemotherapy

## Abstract

Targeted delivery of drugs or other therapeutic agents through internal or external triggers has been used to control and accelerate the release from liposomal carriers in a number of studies, but relatively few utilize energy of therapeutic X-rays as a trigger. We have synthesized liposomes that are triggered by ionizing radiation (RTLs) to release their therapeutic payload. These liposomes are composed of natural egg phosphatidylethanolamine (PE), 1,2-distearoyl-sn-glycero-3-phosphocholine (DSPC), cholesterol, and 1,2-disteroyl-sn-glycero-3-phosphoethanolamine-N-[methoxy (polyethylene glycol)-2000] (DSPE-PEG-2000), and the mean size of the RTL was in the range of 114 to 133 nm, as measured by nanoparticle tracking analysis (NTA). The trigger mechanism is the organic halogen, chloral hydrate, which is known to generate free protons upon exposure to ionizing radiation. Once protons are liberated, a drop in internal pH of the liposome promotes destabilization of the lipid bilayer and escape of the liposomal contents. In proof of principle studies, we assessed RTL radiation-release of fluorescent tracers upon exposure to a low pH extracellular environment or exposure to X-ray irradiation. Biodistribution imaging before and after irradiation demonstrated a preferential uptake and release of the liposomes and their cargo at the site of local tumor irradiation. Finally, a potent metabolite of the commonly used chemotherapy irinotecan, SN-38, was loaded into RTL along with near infrared (NIR) fluorescent dyes for imaging studies and measuring tumor cell cytotoxicity alone or combined with radiation exposure, in vitro and in vivo. Fully loaded RTLs were found to increase tumor cell killing with radiation in vitro and enhance tumor growth delay in vivo after three IV injections combined with three, 5 Gy local tumor radiation exposures compared to either treatment modality alone.

## 1. Introduction

Since the early 20th century, chemotherapy has improved in efficacy but is still mostly a systemic cancer treatment, so patients’ entire bodies are exposed to toxic agents. Non-selective drug delivery leads to marginal accumulation in the tumor and dose-limiting side effects that cause morbidity and can reduce treatment efficacy. Encapsulation of chemotherapeutic drugs in a nanocarrier provides the possibility to increase drug levels in tumors while diminishing side-effects and limiting the impact on efficacy. The main reasons attributed to the limited efficacy of the classic drug delivery carriers are the low accumulation of the drug in tumors and the slow release from the carrier [1]. Tumor-targeted delivery of chemotherapeutics to mitigate devastating side effects and while retaining or improving drug efficacy compared to systemic delivery is challenging but may allow for improved outcomes by using certain drugs to treat primary tumors while using other agents, or reduced systemic dosages, to control systemic micrometastasis. In this regard, many drugs have been incorporated into liposomes, and liposomal drug delivery has shown improved cancer therapy efficacy in some studies [2,3,4]. The delivery of some pH-sensitive drugs is still limited by the instability of the drug molecule itself in a physiological environment or neutral pH [5].

Liposomes generally consist of one or more concentric phospholipid bilayer and an aqueous interior chamber and have the capacity to encapsulate many different types of water-soluble pharmaceuticals [5,6,7]. Liposomes have shown great potential in cancer therapy and therapeutic agent delivery. Most recently, a majority of the successful mRNA-based COVID-19 vaccines were housed in lipid-based nanosize carriers [8,9]. While the current non-triggered release approaches in liposomal drug delivery for the complicated situation of a malignant tumor microenvironment exhibit reduced toxic profiles, some preparations remain associated with systemic drug toxicities [10]. Because radiotherapy is one of the most precise and non-invasive treatment modalities available to treat primary, metastatic, or recurrent tumor volumes, liposomes containing chemotherapeutics that are released by radiation exposure in a spatially and temporally controlled manner represent a major opportunity to overcome toxic sequelae caused by systemic chemotherapy delivery. Radiation-controlled drug release is expected to decrease chemotherapy side effects, increase drug efficacy against tumors, and combine with radiation-induced cell killing. These benefits of radiation-triggered liposomes (RTLs) should allow patients to maintain a higher quality of life along with a higher probability of effective treatment for instance inoperable lung tumors [11].

SN-38, 7-ethyl-10-hydroxycamptothecin, is a highly biologically active metabolite of CPT-11 (Irinotecan) and is a drug with much potential to be used in cancer therapy. However, the clinical use of SN-38 is greatly limited because of its poor aqueous solubility and instability at physiological pH [12,13]. Additionally, the current approaches to administration of SN-38 have resulted in side effects from nausea to more severe adverse reactions such as severe diarrhea and neutropenia [14]. Therefore, SN-38 is a perfect fit in some ways as a drug needing a tumor selective delivery mechanism so that it can avoid normal tissue complications and kill tumor cells with high efficiency before losing activity. Several drug-delivery carriers such as nanoparticles [15,16] liposomes [17] and polymeric micelles [12,18,19] have been envisioned to solve this problem and increase the tumor selective therapeutic effectiveness of SN-38. Among these, liposomes are the most popular, extensively studied, and promising drug-delivery carrier for improving the efficacy of SN-38 in cancer therapy. In fact, liposome irinotecan was recently approved for clinical use [20] and liposomal SN-38 is currently in Phase II clinical trials [21]. However, the formulations do not include a drug-release trigger.

After characterizing the behavior of our patented liposomal formulation under physiological conditions, we worked on a final approach to incorporate the highly toxic SN-38 (7-ethyl-10-hydroxy-camptothecin) into liposomes to improve its chemical stability and enhance its rapid delivery in target specific tissue with prolonged blood circulation time [22,23,24,25,26]. Our initial studies detailed here using the patented radiation-triggered RTL formulation suggest that SN-38 or other drugs with potency against tumor cells, but poor bioavailability or stability can be effectively incorporated into a multi-modality treatment regimen. The potential applications and therapy goals that might be positively impacted by the RTL-mediated approach are discussed.

## 2. Results

### 2.1. Characterization

**Figure 1:**
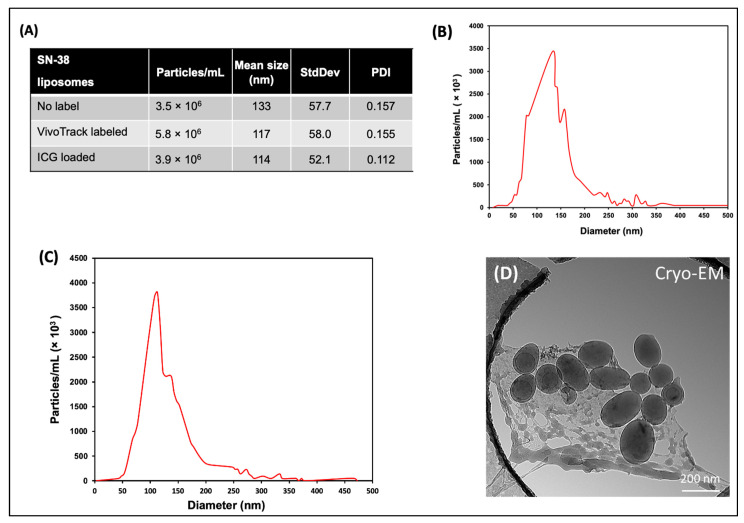
Physical characterization of RTLs produced by extrusion. (**A**) Table of liposome characteristics as measured by NTA at 25 °C. (**B–C**) Representative NTA plots the particle/mL vs. diameter for SN-38 loaded RTLs. (**D**) Cryo-TEM images of RTL liposomes containing PE:DSPC:Chol:DSPE-mPEG (Molar ratio 1:0.5:0.5:0.075).

Nanoparticle tracking analysis (NTA) characterization of the RTL SN-38 loaded liposomes measured a mean size diameter in the range of 114 to 133 nm, from batch to batch (Figure 1A–C), which is in the agreement with the previously reported size range of liposomes prepared by the extrusion method and lipids components with DSPC, PE, Cholesterol, and DSPE-PEG 2000. The distribution of liposome particles in solution were represented by the polydispersity index (PDI), and the value measured at room temperature for fluorophore + SN-38 loaded RTL samples was in the range of 0.112 to 0.157 (Figure 1A). PDI values below 0.3 indicate the homogeneous distribution of lipid vesicles that is acceptable for drug delivery application according to the FDA’s Guidance for industries [27,28,29,30]. Cryo-TEM imaging revealed that the liposomes are well formed, predominantly spherical, and well separated (Figure 1D). The ovoid appearance is likely an artifact of sample preparation.

### 2.2. Payload Release from the Liposomes and Cell Viability

**Figure 2:**
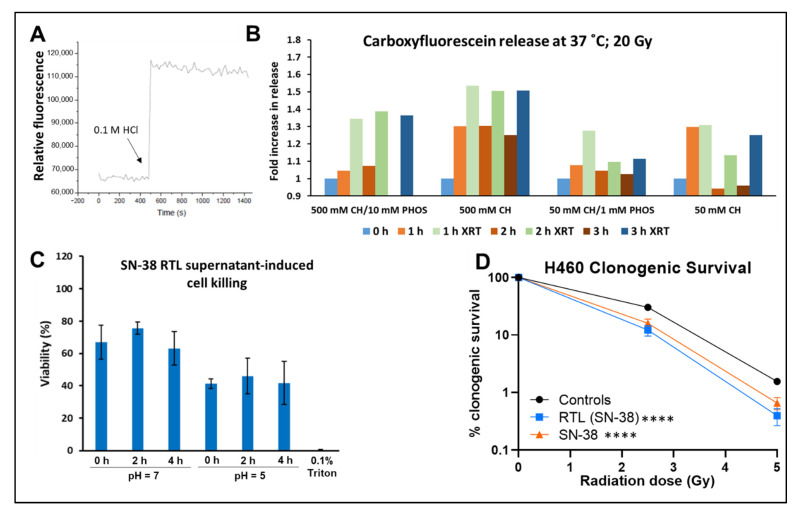
Characterization of in vitro RTL release. (**A**) Demonstration of the pH dependent release trigger as measured by carboxyfluorescein release from RTLs upon acidification with 70 µL of 0.1 M HCl to a final pH of 5.7–5.9. (**B**) Carboxyfluorescein release from RTLs formulated with varying concentrations of chloral hydrate (CH; 50 mM or 500 mM) and phosphate buffer (1 mM or 10 mM) over 3 h of incubation alone or after 20 Gy irradiaton. Data representative of a single experiment. (**C**) Cell viability after exposure to supernatant after incubation of SN-38 loaded RTLs at 37 °C in neutral or acidic pH. Liposomes were incubated in different pH buffers and then supernatant added to the LLC tumor cells. (**D**) Clonogenic survival after incubating cells with SN-38 loaded liposomes or SN-38 alone for 4 h before exposure to 2.5 or 5 Gy of radiation. The drug/liposomes were rinsed off after radiation and replaced with fresh culture medium to allow for surviving cells to form colonies. Average of 3 independent experiments with the bars showing the SD of the mean. Two-way ANOVA with repeated measures for comparison of drug treatments plus radiation vs. radiation alone (**** *p* < 0.0001).

Figure 2 illustrates that we were able to induce release of liposome cargo in vitro by using either and acidic environment (Figure 2A) or radiation exposure (Figure 2B) of liposomes loaded with carboxyfluorescein formulated with varying concentrations of chloral hydrate (CH; 50 mM or 500 mM) and phosphate buffer (1 mM or 10 mM), and then exposed to radiation. Based on the release data in Figure 2B, final RTL formulation for cell viability and in vivo studies include 50 mM CH and 5 mM phosphate buffer. We then incubated SN-38 loaded RTLs in an acidic environment (pH 5) and exposed murine Lewis Lung Carcinoma (LLC) tumor cells to the supernatant (Figure 2C). These results indicate that our trigger mechanism was active under physiological conditions, and we proceeded to study the clonogenic response to combined exposure of H460 human lung tumor cells to drug loaded liposomes with and without radiation exposures (Figure 2D). As illustrated by the survival curves, there was an increased in cell killing when radiation was given in combination with SN-38-loaded liposomes, similar to the effect of free SN-38, suggesting that the release of the drug was occurring upon radiation exposure and either sensitizing the cells to radiation or causing additive cytotoxicity.

### 2.3. Biodistribution

**Figure 3:**
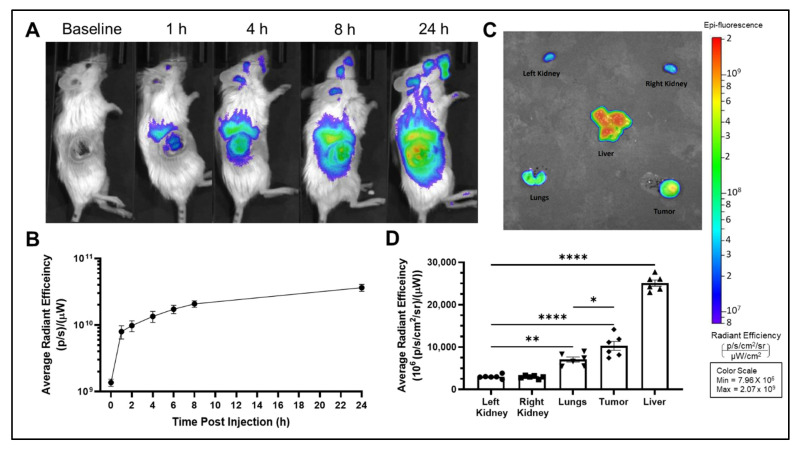
Biodistribution of VivoTrack-labelled RTLs. (**A**) Representative IVIS images of Vivotrack associated fluorescence over the course of 24 h post injection with VivoTrack + SN-38 loaded RTL liposomes. (**B**) Average radiant efficiency in the tumor region of lateral images over 24 h. (**C**) Representative ex vivo IVIS image of VivoTrack associated fluorescence in vital organs and tumor at 24 h post injection of VivoTrack + SN-38 loaded RTL liposomes. (**D**) Average radiant efficiency of vital organs and tumor from a group of six identically treated animals. Data presented as mean ± SEM. One-way ANOVA with Tukey post hoc for comparison of all tissues (* *p* < 0.05, ** *p* < 0.01, **** *p* < 0.0001).

Real-time IVIS imaging revealed that liposomes rapidly accumulated in the tumor by 1 h after injection. Figure 3 shows representative lateral images of VivoTrack-labeled liposomes over time. Rapid tumor accumulation can be seen by the bright signal from the tumor region in the lateral images (Figure 3A) compared to baseline. Relative fluorescence in the tumor region was quantified, corrected for background, and graphed in Figure 3B. The rapid increase from time 0 to the first time point illustrates rapid tumor accumulation of liposomes. Liposomes continued to accumulate in the tumor albeit at a slower rate for up to 24 h after injection, when the experiment was terminated (Figure 3B). Figure 3C shows a representative ex vivo image of the tissues at 24 h post injection and Figure 3D plots the ex vivo fluorescent signal in tissues at 24 h post injection. After normalization for tissue weight, the liver had the highest liposome accumulation followed by the tumor and then the lungs. High liver accumulation is expected based on known liposome biodistribution and lipid metabolism [11]. Additionally, by formulating our liposomes to 100–120 nm in size, we were able to take advantage of the enhanced permeability and retention (EPR) effect seen with tumors [31]. A low accumulation level in the lungs was also observed with these liposomes (Figure 3C,D), which was encouraging as follow up studies will be conducted in orthotopic lung tumor models.

VivoTrack fluorescence in this study only indicates liposome accumulation and does not provide a measure of drug release from liposome. Thus, we observed that VivoTrack-labeled RTL liposomes accumulate in the tumor and liver but did not assess whether they have released their payload into these tissues.

### 2.4. In Vivo Radiation Release

**Figure 4:**
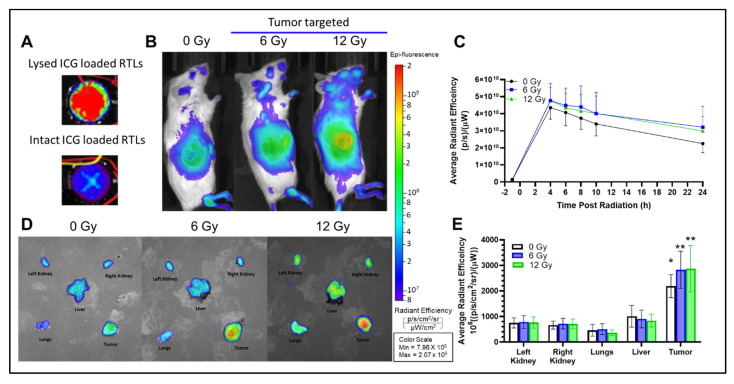
Radiation release of ICG-loaded RTLs. (**A**) Self-quenching ICG loaded liposomes. (**B**) Representative lateral IVIS images of ICG associated fluorescence 4 h post injection and immediately after radiation. (**C**) Average radiant efficiency in the tumor region over the course of 24 h post radiation. (**D**) Representative ex vivo IVIS imaging of ICG associated fluorescence in vital organs and tumor at 24 h post radiation. (**E**) Average radiant efficiency of vital organs and tumor. Data presented as mean ± SEM; *n* = 5–6. One-way ANOVA with Tukey post hoc for comparison of all tissues to tumor for each group (* *p* < 0.05, ** *p* < 0.01).

ICG was loaded into liposomes at a self-quenching concentration (0.25 mg/mL). Thus, fully intact liposomes displayed minimal fluorescence (Figure 4A). Based on biodistribution data in Figure 3B, 4 h was determined to be the optimal time after liposome injection to administer radiation. This timepoint would provide sufficient liposome accumulation in the tumor and allow for serial imaging post radiation. Figure 4B shows representative images at 4 h after injection with quenched ICG loaded liposomes alone or in combination with 6 or 12 Gy tumor-targeted radiation. The left mouse received no radiation, middle panel received 6 Gy tumor irradiation just before imaging, and right panel received 12 Gy tumor irradiation just before imaging. There was evidence of enhanced ICG signal in four out of six irradiated tumors compared to non-irradiated tumors indicating that the ICG had been released into the tumor microenvironment. The relative fluorescence in the tumor region was quantified, corrected for background, and plotted in Figure 4C. The ex vivo images taken of tumor and major organs also agreed with this trend. Figure 4D shows a representative ex vivo image of the tissues from all three groups. Figure 4E shows ex vivo fluorescent signal in tissues at 24 h post injection. After normalization for tissue weight, the tumor had the highest liposome cargo found for all three groups. There is a small increase in liposome release in the tumors of irradiated mice vs. unirradiated mice, but this difference was not statistically significant. However, minimal ICG fluorescence was detected in any other tissue type. Importantly, the timeline for image collection in Figure 4 is time since radiation, as opposed to the biodistribution study (Figure 3) which was measured as time since injection. Therefore, the timepoints for Figure 4C are shifted to the right by 4 h. Additionally, the difference in fluorophore wavelength and detection between this study and the previous biodistribution study could affect background and accumulation of signal, and therefore we cannot make direct comparisons between these two studies.

**Figure 5:**
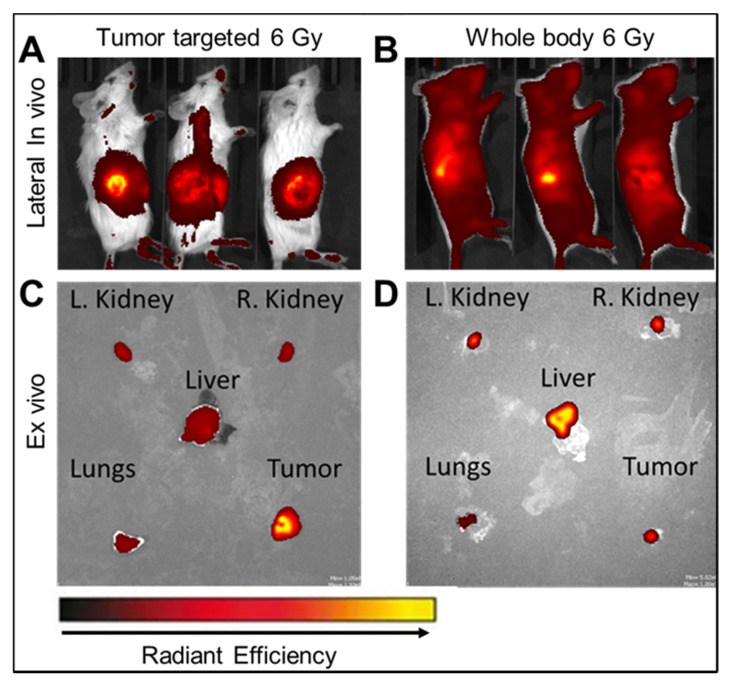
ICG release from RTLs after tumor-target or whole-body radiation. Representative lateral IVIS images of ICG associated fluorescence 4 h post injection and immediately after (**A**) tumor targeted radiation or (**B**) whole-body radiation. Representative ex vivo IVIS imaging of ICG associated fluorescence in vital organs and tumor at 24 h post (**C**) tumor targeted radiation or (**D**) whole-body radiation. Images representative of *n* = 6.

Importantly, compared to tumor-targeted radiation (Figure 5A), when mice were treated with whole-body radiation, a strong ICG fluorescent signal was detected in all tissues (Figure 5B). Compared to the ex vivo imaging in tumor targeted radiation (Figure 5C), liposome release detected in whole-body radiation was significantly higher in the liver, lungs, and kidneys (Figure 5D). In particular with whole-body radiation, the liver displayed the highest liposome release (Figure 5D), which corresponds to the biodistribution study where the liver displayed the highest liposome accumulation.

### 2.5. Anti-Tumor Efficacy

**Figure 6:**
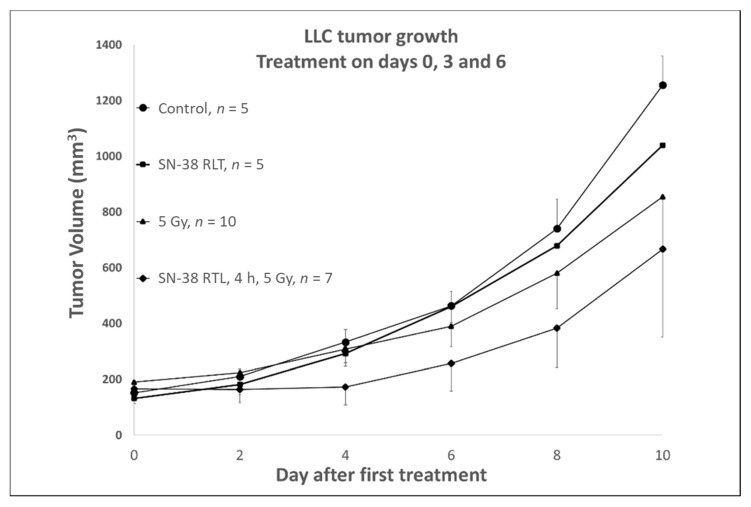
Tumor growth delay compared to untreated (control) LLC tumors grown in the rear limb of C57 mice. Groups contained 5–10 individually treated mice and the data is plotted as the mean with SD. SN-38 2 mg/kg or RTLs loaded with SN-38 corresponding to 2 mg/kg were injected IV on days 0, 3 and 6. Radiation was administered to the tumor at a dose of 5 Gy on days 0, 3 and 6. In the combined treatment group, RTLs (SN-38) were given IV at 2 mg/kg and 4 h after injection the tumor was irradiated with 5 Gy. The average tumor volume at days 2–8 after the start of treatment in the combined therapy group was significantly lower (*p* < 0.0002–0.04, Student’s *t*-test comparing average fold change in tumor volume) than either monotherapy or control group. By day 10, the last day of measurement, the tumors had all started to regrow at a similar rate since therapy had ended on day 6.

A tumor growth delay assay was performed to assess whether or not the combined treatment strategy as designed and tested could produce an increase in the tumor control. As shown in Figure 6, we studied the combined effect of SN-38 loaded RTLs alone, 5 Gy alone, or SN-38 loaded RTLs followed by 3 fractions of 5 Gy on Lewis lung carcinoma (LLC) growing in the rear limb of mice. No significant effect was found with SN-38 RTLS alone on tumor growth compared to untreated controls, while the addition of liposomal SN-38 before tumor irradiation was found to clearly enhance the tumor control during and just following the drug and radiation fractionation (~6–8 days), suggesting that significantly improved tumor control may occur with repeated radiation and locally released drug, as well as more selective tumor effects judging from the biodistribution and radiation-release data that we have obtained (Figure 3, Figure 4 and Figure 5).

## 3. Discussion

The field of drug delivery and targeted release of therapeutics using nanomaterial-based vehicles has a long history, dating back at least 50 years to the early work of Gregoriadis [32,33]. While a wide variety of materials have been studied and investigated as drug carriers, the liposome has garnered the most attention. Yet, the true control and design of many lipid-based drug delivery systems remains less than optimal. Our goal in using radiation therapy as a trigger for drug release was to exploit one of the most non-invasive, potent, and precise therapies available to the oncologist. Radiation allows control and precise tumor volume targeting while avoiding significant triggering in any normal tissues, by virtue of the precision and accuracy of modern-day radiotherapy treatment planning. There have been extensive efforts with triggering mechanisms using thermal therapy, with some success [34]. However, in most cases the control of the trigger timing and tissue volume of effect remains inexact due to the inherent nature of blood flow, thermal washout from tissues and other vascular permeability issues. While these will also be factors in our current platform, we surmise that the control and precision by which we can activate release from liposomes taken into the interstitial space or into the tumor cells themselves will facilitate a better therapeutic index that other approaches. By using conformal radiotherapy that does not depend on blood flow or conduction for dose deposition we add a new level of control which warrants more development and testing.

This report summarizes the initial studies undertaken by the company/university team at our institutions. Herein we have shown that our patented liposomes can be formulated to the size range and polydispersity index considered acceptable for drug delivery applications by the FDA’s guidance for industries [27,28,29,30]. Moreover, we demonstrated that the chemotherapy agent, SN-38 can be encapsulated in these liposomes, while maintaining the pH sensitivity that underlies the triggered release mechanism and cytotoxic actions of the drug. This is evidenced by our cell viability and clonogenic assays that show decreased survival with addition of just the supernatant after liposomes were exposed to low pH or ionizing radiation. We interpret this to mean that robust quantities of active SN-38 were released from the liposomes after triggering the release mechanism. It is this delicate chemical interplay between pH, liposome stability, and drug action that makes our formulation stand out from other liposome preparations.

The rapid tumor uptake and retention of our RTLs illustrated the enhanced permeability and retention effect [31], which is a universal pathophysiological phenomenon whereby macromolecular compounds such as proteins, lipid vesicles and polymer-conjugated drugs above 40 kDa progressively accumulate in the tumor vascularized area due to the “leakiness” of the newly formed blood vessels around the tumor. Properly sizing our liposomes between 100–120 nm allowed us to take advantage of this phenomenon and achieve selective tumor delivery and retention. One aspect of our liposome platform that is also at work is the pH sensitivity of our lipid composition. While we have initially found that radiation induces a rapid and large enough change in pH to release the liposome cargo, we also recognize that the low pH of the tumor microenvironment itself may be inducing greater release of the drug/tracer molecules than in other tissues that maintain a more neutral pH. This can be seen in the data shown in Figure 4D,E where even the tumor tissue that was not irradiated was found to have higher levels of the loaded ICG than in most other tissues. We interpret this to reflect the induced release of cargo by the liposomes that were taken up in the acidic tumor microenvironment. It is this mechanism that most pH sensitive liposome formulations intend to harness to promote tumor selective drug delivery. However, in the absence of a release trigger these pH sensitive liposomes release their payload slowly, which may limit maximum drug concentrations at the tumor, whereas the release from our RTLs was found to be further enhanced and more rapid once local radiation was administered. These combined release factors are likely what led to the increased tumor response found in the growth delay study shown in Figure 6. While the rapid growing tumor model used in our proof of principle studies here did not allow for a lengthier course of liposome and radiation treatments, an indication of treatment synergism was observed over the course of six days and three combined treatments where the tumor growth was clearly inhibited or at the least altered compared to the single treatment modalities or tumors that were left untreated. Compared to the single radiation dose (6 Gy) used in the radiation release studies, we modeled our anti-tumor efficacy treatment as a fractionated radiation regimen to assess the efficacy of multiple rounds of treatment with drug containing liposomes. This radiation dose (5 Gy) was chosen to limit the total radiation dose (15 Gy) and still provide sufficient triggered liposomal release. The tumor volume was maintained at a significantly lower size for a week (days 2–8 after treatment) which suggests that had we continued treatment, as would be the case in a clinical version of this treatment modality, we could have achieved far greater tumor control. These are all indications that warrant further expanded studies to prepare our liposomal platform for translation, either with the current drug (SN-38, nearing FDA approval) or with other already approved agents that could be easily brought into currently accepted chemoradiation regimens for a number of disease sites.

Additionally, our RTLs include PEGylated lipids and are formulated to be “stealth” liposomes, which evade uptake and breakdown by macrophages and Kupffer cells in the liver [35]. While our RTLs accumulated in tissues outside the tumor region including the liver; there appeared to be minimal release or retention of SN-38 without the radiation trigger. These findings suggest that our liposomes may be able to deliver tumor-targeted cancer therapy with minimal exposure to other tissues. All in all, we have provided proof of concept that our drug delivery platform, RTLs, can provide tumor-targeted drug release when combined with radiation, and thus has the potential to enhance tumor control while reducing off-target toxicities. These findings raise the possibility of encapsulating a variety of chemotherapeutic agents to improve their bioavailability, stability, and tumor uptake, while limiting exposure to other tissues. However, it is possible that the protocol and buffering agents may need to be modified to successfully encapsulate other chemotherapeutics based on their unique chemistries. Future studies will focus on pharmacokinetics and more extensive dose response studies to further evaluate tissue drug concentration, elimination half-life, anti-tumor effectiveness, and off-target toxicities.

## 4. Materials and Methods

### 4.1. Chemicals

1,2-distearoyl-sn-glycero-3-phosphocholine (DSPC), 1,2-distearoyl-sn-glycero-3-phosphoethanolamine-*N*-[methoxy (polyethylene glycol)-2000] ammonium salt (DSPE-m-PEG2000 or 18:0PEG2000PE), and cholesterol were bought from Avanti Polar Lipids LLC (Alabaster, AL, USA), L-α-Phosphatidylethanolamine (PE) from egg yolk was purchased as a lyophilized powder from Sigma Aldrich (St. Louis, MO, USA). All lipids purchased were used without further purification. SN-38 in powder was purchased from Fisher Scientific (Waltham, MA, USA) and its stock solution was prepared in dimethyl sulfoxide (DMSO; 10 mg/mL). Reagents such as methanol and chloroform were purchased from Fisher Scientific and used as provided. Sephadex G-75 and carboxyfluorescein were purchased from Sigma Aldrich (St. Louis, MO, USA). Indocyanine green (ICG) was purchased from Fisher Scientific (Waltham, MA, USA). Vivotrack 680 was purchased from PerkinElmer (Hopkinton, MA, USA).

### 4.2. Animals

For biodistribution and tumor radiation release imaging studies, 8–12-week-old female C57BL/6J albino mice were purchased from Jackson Laboratories (Bar Harbor, ME, USA). Mice were inoculated subcutaneously in the flank with 1 × 10^6^ Lewis lung carcinoma cells (LLCs; ATCC, Manassas, VA, USA). For tumor efficacy study, 8–12-week-old male C57BL/6J mice were purchased from Jackson Laboratories (Bar Harbor, ME, USA). Mice were inoculated subcutaneously in the rear limb with 1 × 10^6^ Lewis Lung Carcinoma (LLC) cells. Experiments were started 7–10 days after tumor inoculation when the tumors reached a volume of 250 mm^3^. All procedures were carried out in accordance with the Guide for the Care and Use of Laboratory Animals as adopted and promulgated by the U.S. National Institutes of Health and approved in animal use protocols at Charles River Laboratories and by the Institutional Animal Care and Use Committee at the University of Arkansas for Medical Sciences.

### 4.3. Liposomes Preparation

#### 4.3.1. Preparation of Spherical Lipid Bilayers

The lipid components used to prepare lipid bilayers composed of PE, DSPC, Cholesterol, and DSPE-mPEG (Molar ratio 1:0.5:0.5:0.075) and prepared by the extrusion method as described previously [21]. Briefly, lipids with a combined mass of 20.5 mg were dissolved in 1.3 mL of chloroform in a glass vial. The homogeneous mixture of lipids was then transferred to a 50 mL round bottom centrifuge tube using a Hamilton syringe. A dried thin film lipid bilayer was formed in the tube by rotatory evaporation of solvent under vacuum at 50 °C at a constant speed of 100 rpm for 2 h.

#### 4.3.2. SN-38 Loaded Liposome Preparation

The tube was warmed at 70 °C for 30 min and the lipid bilayer was disrupted by adding 27 mm glass beads to the tube and shaking continuously. The thin film lipid bilayer was hydrated with 1.3 mL of saline solution (pH 7.2–7.4) containing 50 mM Chloral hydrate, 5 mM phosphate, and 1 mg/mL SN-38. Approximately 120 nm size SN-38-loaded liposome was obtained by passing the suspension through a mini-extruder (Avanti Polar Lipids) using polycarbonate filter membranes with pore sizes 400 nm, which was further downsized by a 100 nm pore size filter membrane. The unencapsulated SN-38 was removed using a Sephadex G-75 column equilibrated with 0.9% saline solution (pH 7.2).

#### 4.3.3. Carboxyfluorescein- and ICG- Loaded Liposome Preparation

Carboxyfluorescein- and ICG (indocyanine green)-loaded liposomes were prepared in a similar way as described above for the fluoresence release and in vivo animal studies. Carboxyfluorescein and ICG were dissolved in PBS adjusting pH to 7.4 using 0.1 M NaOH or 0.1 M HCl. Carboxyfluorescein was loaded into the liposome at the concentration of 30 mM whereas ICG was at 0.25 mg/mL. The stock solutions of these fluorescent dyes were prepared in the PBS and added to the loading solution at desired concentration prior to the extrusion of lipids suspension. The excess carboxyfluorescein or ICG from the liposomes were removed using a Sephadex G-75 column equilibrated with 0.9% saline solution (pH 7.2). Empty liposomes were prepared in a similar way as described above except for the addition of SN-38, carboxyfluorescein or ICG into the loaded solution. All the liposomes prepared were stored at 4 °C for future use.

#### 4.3.4. Liposome Labeling with Vivo-Track 680

NIR fluorescent VivoTrack dyes were used for in vivo imaging. In this paper, liposomes labeling with Vivotrack-680 was performed as follows. First, the VivoTrack-680 solution in PBS was prepared according to the Technical Data Sheet provided by the PerkinElmer Company (Hopkinton, MA, USA). In brief, 0.2 mg of VivoTrack-680 powder was dissolved in warm sterile 1.3 mL of 1× PBS by gentle swirling and vortexing to yield 2.0 mL of the labeling agent (0.1 mg/mL). After ensuring the solid was fully dissolved in the PBS solution, it was stored at 4 °C protected from the light with aluminum foil for labeling with liposomes. Next 1 mL of the prepared liposome loaded with SN-38 was ultra-centrifuged at 137,000× *g* for 30 min, the supernatant was discarded, and 1 mL of the VivoTrack-680 solution was added to liposome pellet. The solution was mixed immediately by vortexing and incubated for 15 min at room temperature protecting from the light. After incubation, the solution was washed 3 times by ultracentrifuge at 13,700× *g* for 30 min each time with 1 mL of PBS to remove the excess of labelling agent. The final liposome pellet with VivoTrack-680 labeling agent was resuspended in 1 mL PBS.

### 4.4. Characterization

#### 4.4.1. NTA (Nanoparticle Tracking Analysis)

The hydrodynamic diameter, particle size distribution/mL, and particle concentration/mL of liposomes were determined using Nanoparticles Tracking Analysis (NTA; Particle Metrix (Ammersee, Germany), ZetaVIEW S/N 18-379, software ZetaView 8.04.02 SP2, Camera 0.713 µm/px). Samples were diluted in a ratio of 1:100, 1:1000, 1:10,000, 1:100,000, 1:100,000 in PBS and ran in the instrument. One mL of the diluted sample was loaded into the 1 mL plastic syringe, injected into the instrument, and the measurements carried out upon illumination by the laser light (635 nm).

#### 4.4.2. Cryo-Electron Microscopy (Cryo-EM)

The surface morphology, shape, and size of the liposome were frozen using a Leica EMPACT2/AFS2 (Wetzlar, Germany) and sliced by a Leica Ultracut 7 Microtome (Wetzlar, Germany) with Cryo-attachment. Samples were then observed using a FEI Tecnai F20 200 keV Transmission Electron Microscope (TEM) in the University of Arkansas for Medical Sciences Digital Microscopy Core.

#### 4.4.3. Release of Carboxyfluorescein

Liposomes were loaded with 50 mM carboxyfluorescein in saline solution containing 50 mM or 500 mM Chloral hydrate alone or with 1 or 10 mM phosphate. RTLs were then incubated at 37 °C and a portion was irradiated with 20 Gy at the 0 h. At 1, 2, 3 h post irradiation a sample was taken out and centrifuged at 137,000× *g* for 30 min and transferred to 96 well plate, where the fluorescence signal was measured at excitation/emission range of 490/517.

### 4.5. In Vitro and In Vivo Efficacy

#### 4.5.1. Cell Viability

SN-38 loaded liposomes were incubated at 37 °C in neutral and acidic saline solution (pH adjusted to pH 7 and 5 using 10 mM Citrate buffer) for 0, 2, and 4 h, and then centrifuged at 137,000× *g* for 30 min. 5 µL of the supernatant was added to 100 µL of medium in 96 well plate containing LLC tumor cells. As a control for cell killing effect of full release of SN-38, cells were treated with supernatant from liposomes treated with 0.1% Triton X-100. Cell viability was assessed using CCK8 method and values were normalized to the relevant untreated controls.

#### 4.5.2. Clonogenic Assay

H460 human lung tumor cells were treated with free SN-38 or SN-38 loaded liposomes for 4 h and then subjected to graded radiation of 2.5 and 5 Gy. Fifteen minutes after radiation media was removed and replaced with fresh media. Cells were incubated for 5–7 days to form colonies and then stained with crystal violet blue, colonies were counted, and normalized to the untreated controls of each treatment to evaluate the survival.

#### 4.5.3. Biodistribution

Eight- to twelve-week-old female C57BL/6J albino mice were inoculated subcutaneously in the flank with 1 × 10^6^ LLC cells. Tumors were allowed to grow for 7–10 days until they reached a target size of 250 mm^3^. A baseline whole-body ventral and lateral image was captured using an IVIS imager (PerkinElmer, Hoptkin, MA, USA). Then, animals were administered VivoTrack–labeled, SN-38-loaded RTL liposomes (100 µL) and whole-body lateral images were collected at 1, 2, 4, 6, 8, and 24 h post injection. Immediately following the 24 h image vital organs (liver, whole lung, kidneys) and tumor were collected and imaged ex vivo. Vivotrack associated fluorescence in the tumor region of the whole-body images was analyzed for each time point and compared to baseline to determine time to optimal tumor accumulation for the subsequent radiation release study. Vivotrack associated fluorescence in vital organs and tumor were used to determine relative RTL biodistribution.

#### 4.5.4. Radiation Release in the Tumor

Eight- to twelve-week-old female C57BL/6J albino mice were inoculated subcutaneously in the flank with 1 × 10^6^ LLC cells. Tumors were allowed to grow for 7–10 days until they reached a target size of 250 mm^3^. A baseline whole-body lateral image was captured using an IVIS imager. Then, animals were randomized into 4 groups and were administered either, ICG-loaded RTL liposomes (100 µL), ICG loaded RTL liposomes (100 µL) +6 Gy tumor targeted radiation, ICG loaded RTL liposomes (100 µL) +12 Gy tumor targeted radiation, or ICG loaded RTL liposomes + 6 Gy whole-body radiation. Animals were injected intravenously with RTL liposomes first and then irradiated 4 h after injection. Whole-body lateral images were collected at 0.5, 2, 4, 6, and 24 h post irradiation. Immediately following the 24 h image vital organs (liver, whole lung, kidneys) and tumor were collected and imaged ex vivo. ICG associated fluorescence in the tumor region of the whole-body images was analyzed for each timepoint and compared to baseline to determine liposome release. ICG associated fluorescence in vital organs and tumor were used to determine relative liposomal release. Irradiation was performed using 225 keV beam generated by a Faxitron MultiRad 225 X-ray system (Hologic, Tuscon, AZ, USA).

#### 4.5.5. Anti-Tumor efficacy

Eight- to twelve-week-old male C57BL/6J mice were inoculated subcutaneously in the rear limb with 1 × 10^6^ Lewis Lung Carcinoma cells. Tumors were allowed to grow for 7–10 days after tumor inoculation until the tumors reached a target of 250 mm^3^. Then, animals were randomized into 5 groups and were administered saline, free SN-38, SN-38 loaded RTL liposomes, 5 Gy radiation, or SN-38 loaded RTL liposomes + 5 Gy radiation. Animals were dosed every 3 days for a total of 3 cycles. For animals receiving combination therapy, SN-38 loaded liposomes were injected first and then tumors were irradiated 4 h post injection. Tumors were measured with calipers on Day 1 and then every 3 days. Irradiation was performed using 320 keV beam generated by XRad320 (Precision X-Ray, Branford, CT, USA).

## 5. Patents

The liposomes studied in this manuscript are protected by a United States patent US 9,849,087 B2 and European Patent EP2776013A1.

## Data Availability

The data presented in this study are available on request from the corresponding author.
